# Cases of Emphysema of the Lungs at the Louisville Marine Hospital, with Remarks

**Published:** 1854-05

**Authors:** Austin Flint

**Affiliations:** Professor of the Theory and Practice of Medicine in the University of Louisville


MEDICINE AND SURGERY.

MAY, 1854. NEW SERIES. VOL. I.—NO. 5.

### ART. I.—CASES OF EMPHYSEMA OF THE LUNGS AT THE LOUISVILLE MARINE HOSPITAL, WITH REMARKS.

BY AUSTIN FLINT, M. D., PROFESSOR OF THE THEORY AND PRACTICE OF MEDICINE IN THE UNIVERSITY OF LOUISVILLE.

THREE CASES of Emphysema of the lungs have lately been presented before the medical class at the Amphitheatre connected with the Louisville Marine Hospital. This affection, interesting in itself, has pathological relations of great interest and importance. It is an affection by no means very common, yet, on the other hand, not extremely rare. There is reason to believe that it is often overlooked, or insufficiently appreciated by practitioners of medicine. In view of these considerations, I propose, in this article, to give a report of these three cases, respectively, embracing an account of the symptoms and physical signs at the dates of admission and of discharge, and appending a few general remarks which the facts contained in the histories of the cases will suggest.

In each of the three cases the disease was of that form called *vesicular* or *true Emphysema*, the morbid condition, in this variety of the disease, consisting in a permanent enlargement, in a greater or less degree, of the *air vesicles* of the lungs, either by distension, or rupture of the cell walls and coalescence, or both being combined; differing from what is distinguished by the name of *interlobular Emphysema*, in that the latter consists of the extravasation of air into the areolar structure interposed between the pulmonary *lobules*. Interlobular Emphysema is undoubtedly a veritable affection, but so rare that, practically, it possesses not much importance. It is safe to say that ninety-nine of one hundred cases of emphysema presenting themselves in practice, are of the kind called *true* or *vesicular*.

An important distinction pertains to the point of departure, or the primary changes of structure in different cases of vesicular emphysema, which will account for some variations in the physical signs, and may explain certain discrepancies appearing in the writings of different observers. In some instances the affection commences with, and becomes developed in consequence of atrophy and dissolution, more or less of the intervesicular septa. As the result of this atrophy and dissolution, coalescence of the cells, to a greater or less extent, takes place. The area of the respiratory surface, of course, is diminished in proportion to the extent of coalescence; just as the interior surface of a single hollow globe is less than the aggregate of the inner surfaces of a series of smaller globes. Here the point of departure, or the primary change, is the destruction of the partition walls of the air cells. In this species of emphysema the volume of the lungs may be but little increased. This is an important point of difference with reference to the external appearance of the chest, and some of the physical phenomena. As emphysema thus developed is especially incident to advanced life, it is sometimes known as *senile emphysema*.

In other instances the disease commences with an abnormal and permanent enlargement of the cavities of the cells. They become over-distended, and, losing more or less of their elasticity, they continue in this condition. It is thus a kind of wind dropsy. There is an excess of air in the cells, but it is retained there, and inasmuch as the chemical changes to be wrought in the blood require constantly fresh supplies of air, as of blood, the effect is precisely the same as if the cells were diminished in size to a degree rendering their capacity insufficient for the function of hematosis. Over-distension is the point of departure in the primary change, in this second kind of emphysema, and in this variety of the affection the volume of the luugs, or of the portions affected, is notably increased, and, hence, greater enlargement of the chest, and other phenomena dependent on the larger quantity of air which the lungs contain, and the greater pressure exerted on the thoracic walls. In this latter kind of emphysema, however, it is to be remarked, atrophy of the intervesicular septa may occur, but in this instance, it is a secondary, not a primary lesion. It will be seen that the cases which are to follow illustrate the form of Emphysema last mentioned.

Both kinds of Emphysema, so far as regards the relation of the disease to its important symptoms, and effects, are identical; in other words, after the affection becomes developed, the pathology is the same. The train of evils, in either instance, arises, first, from defective performance of the blood changes dependent on respiration, and second, from the obstacle which is presented to the free transmission of blood through the pulmonary capillaries. The diminished area of the respiratory surface of the lining membrane of the cells, in consequence of destruction of the walls and coalescence, and the inability to replace sufficiently, by successive acts of respiration, the air contained in the cells, account for the defective blood changes. The obstacle to the free circulation through the pulmonary capillaries arises from several causes. *First*, the capillaries are diminished in proportion to the destruction of the cell walls; *Second*, the over-distension compresses the vessels and thus becomes a source of resistance to the passage of blood; and, *Third*, inasmuch as the chemical actions going on between the blood and atmosphere develop an attractive force which helps to carry on the pulmonary circulation, a morbid diminution in these changes impairs proportionately this attractive force, and leads to a retarded flow of blood. In consequence of the obstacle to the pulmonary circulation, thus, in different ways incident to emphysema, the right ventricle suffers an over-accumulation of blood. As consequences of this result we observe some symptoms in cases of the disease referable to a congested state of the systemic venous system; and, as a remote effect, we find enlargement of the heart, sooner or later, occurring, the evils of which are added to those more directly due to the condition of the lungs.

With these few preliminary remarks, I proceed to give, from the hospital records, an account of the cases, commencing with the case in which the emphysematous lesions were least in degree, and ending with the one in which the characters of the affection are the most strongly marked.

---

CASE 1.—*Michael Fanning*, aged thirty-seven, Irish, machinist, admitted December 22d, 1853.

*Previous History.*—States that he is not aware of any of his family having been affected similarly to himself. At about the age of sixteen years, he began to have paroxysms of difficulty of breathing, occurring generally at night, requiring him to take the sitting posture. From that time to the present has generally been conscious of a sense of labor in respiration. For the past twenty years has not been able to take active exercise without a sense of breathlessness. Whenever he has contracted cold, the embarrassment of respiration has been considerable, obliging him to quit work for a time. His respiration has always been better in the summer than in the winter. The habitual sense of labor in respiration he thinks was greater for the first five years, than since. He is not aware that his malady has increased for the last fifteen years in that respect, but he has found that he cannot bear active exercise as well as formerly, experiencing therewith greater want of breath. He has been more subject to colds of late years than formerly, and they are attended with greater disturbance of respiration. Has been subject for the past twenty, or more, years to paroxysms of difficult breathing, occurring without cold, exercise, or any obvious cause. These paroxysms are very irregular in their recurrence, being most apt to come on during the night, but sometimes in the day time. They have occurred, more or less, weekly, and sometimes several times in the twenty-four hours. They last from five minutes to an hour. They have increased in frequency and severity every year. He prefers to lie with his head elevated. He cannot lie on a feather bed; he says it "stops his breath" to do so, and that he is obliged to get up and lie on the floor. He has had more or less cough and expectoration for the last twenty years. These symptoms have increased, but not greatly. Has never had hemoptysis, hut has frequently expectorated mucus streaked with blood. He thinks he has preserved the same weight for several years past. During the last summer, while affected with intermittent fever and diarrhœa, he lost flesh, but has gained, in that respect, of late. Has worked, more or less, during this whole period. He has been accustomed to drink daily for the last sixteen years, and has occasionally drank to excess.

*Present Condition.*—Laboring under an attack of bronchitis. Face presents a puffy appearance, and is deeply congested. Suffers much from paroxysms of coughing attended by dyspnœa, especially during night. Expectorates freely muco-purulent matter. Appetite poor. Has slight diarrhœa, with some tenderness over abdomen, and tympanites. He is not confined to the bed, and is not emaciated. Pulse 114, very feeble and quite irregular. Respirations 24. They are labored, and the labor is especially marked in the expiration. He is not at the time of this examination laboring under a paroxysm of difficult respiration.

*Physical Signs.*—The chest is full and rounded, the right side more so than the left; the clavicles are but slightly apparent. The expansive movement is slight. Respiration is diaphragmatic, the abdominal muscles acting with expiration. The upper part of the chest inclines forward.

*Percussion.*—Both sides, in front, yield a very clear percussion sound. The left side gives a fuller and somewhat graver note than the right. The sense of resistance is distinctly greater on the right side. Hepatic flatness commences with the sixth rib, and on line of nipple. Clear percussion sound on both sides behind.

*Auscultation.*—There is so little movement of the upper part of the chest with respiration, that it is somewhat difficult with the ear applied to the chest, to tell with which of the respiratory acts the sounds correspond. They appear to be sometimes with one, and sometimes with the other.

On examination at the time of noting the signs, there is a feeble, shortened sound of inspiration on the right side, at the summit of the chest, and an obscure râle accompanying expiration. On the left side the inspiratory sound is better evolved, and longer, with no distinct sound of expiration, but an obscure râle taking its place.

Over the middle and lower thirds, on both sides, a faint inspiratory murmur is alone heard. Behind very faint inspiration over scapula, and scarcely appreciable below.

Vocal resonance slight, and about equal on both sides, at the summit, in front.

Vocal fremitus slight and about equal on the two sides.

Apex impulse of heart not appreciable by touch, but faintly visible at the nipple. Relative dullness transversely, in precordia, extends to about an inch beyond nipple. Heart sounds very feeble but pure.

*Treatment.*—Anodyne, expectorant mixture, and the application of croton oil to chest. Nutritious, non-animal diet.

January 15th, 1854. The patient has progressively improved to this date, and now feels sufficiently well to be discharged. The diarrhœa has ceased. Appetite is good. Cough much less troublesome, and expectoration small. The respiration is less labored than when he entered the hospital, but active exertion occasions panting as heretofore. Pulse 88, and frequently intermitting. Respirations 28. Aspect pallid, but no lividity. Expression does not denote anxiety. No morbid change in countenance except pallor.

*Physical Signs.*—Form of chest not notably altered. The infra clavicular and post clavicular spaces are not depressed. The clavicles do not project, their outline simply being apparent. The spaces, however, above and below the clavicle are not bulging. The right infra clavicular region is fuller than the left. Intercostal depressions are no where visible, except at the lower and lateral portions of the chest. On a posterior view no intercostal depressions are apparent. The patient presents moderate *embonpoint.* The movements of the chest in respiration are very slight. The expansive movement at the summit, on the right side, is almost null. On the left side, slight. An evident disparity exists between the two sides in this particular. The entire chest presents, with inspiration, a moderate elevation movement, except at the lower portion, and here it appears slightly to contract. Behind, the upper part of the chest inclines forward. The spine is curved, first backward and then forward. The right scapula moves with the respirations more than the left. On forced respiration, the whole chest is elevated as if in one piece, the elevation movement becoming conspicuous, while the expansive movement is not increased. Walking briskly twice across the road occasions panting respiration with the characters just described. The heart's action is also thereby increased.

An impulse of the heart cannot be felt while he is tranquil, but after walking across the ward it is indistinctly felt by the side of the ensiform cartilage, and not elsewhere.

The expiratory acts are evidently longer than the inspiratory.

The percussion sound is very resonant at the summit of the chest on both sides. On the right side it is more tympantic. The pitch on the right side is slightly higher. The resistance of the thoracic wall on the right side is in a marked degree greater than on the left. Posteriorly, over the scapula, the percussion sound is abnormally clear.

In the precordial region the sound is quite dull transversely as far as the nipple.

No respiratory sound is appreciable anywhere save an indistinct murmur with the inspiration in the right interscapular space.

Neither vocal resonance, nor fremitus are distinctly appreciable.

On mensuration the right side is seventeen and three quarter inches; and the left, seventeen inches.

The sounds of heart full and pure.

Patient discharged.

---

Case 2.—*Mary Jane Kesterson*, aged nineteen, widow, admitted November 27th, 1853.

*Previous History.*—States that she enjoyed tolerably good health prior to about two years ago, when she had measles. Since that time she has suffered constantly, more or less, from cough, and difficult respiration. During the past two years has never been free from a sense of labor in respiration, but experiences much greater difficulty at some times than at others. She has been subject to paroxysms of dyspnœa coming on irregularly, and also irregular as respects duration. These paroxysms are most apt to occur at night, and frequently she has several in succession during the same night. She also suffers similar attacks during the day time. Exercise occasions difficulty of breathing. Laughing has the same effect, with acts of coughing, when violent, she feels as if she should *lose* her breath.

*Present Symptoms.*—She has a pretty large expectoration, decidedly purulent, but the quantity, she says, is less than heretofore. Face presents a puffy appearance, with some lividity of prolabia. Sleeps best when she lies with her face downward. The acts of coughing are violent and spasmodic, resembling the coughing in pertussis, exclusive of the sonorous inspiration, or whoop, of that affection. During a paroxysm of coughing, the face becomes deeply congested, and the respiration greatly embarrassed. Expectorates with considerable effort, although the matter of expectoration is not adhesive. She has slight dyspnœa constantly. Her skin is cool and dry. There is moderate capillary congestion of the upper extremities. She suffers from cold hands and feet. The radial pulse is thread-like and so as to be enumerated with difficulty. It is 100 per minute. Respirations 30, and moderately labored when the paroxysmal difficulty is not present.

The patient has had one child, not living. Has no knowledge of her parents or any of her progenitors having been affected like herself.

*Physical Signs.*—Nov. 30.—The anterior superior portion of the chest, on both sides, is full and rounded, the left side is fuller than the right. There is considerable elevation movement with inspiration, but the expansive movement is slight. The whole superior part of the chest, including the clavicle, is elevated with inspiration. The right side expands more than the left, and is evidently more depressed with expiration. The lower portion of the chest appears contracted, and seems to be drawn in with the acts of inspiration.* The expiratory act is in a marked degree longer in duration than the inspiratory. It is at least twice as long. This is, in part, due to abnormal shortness of the inspiration. On comparing the appearance of the chest to day, with that of yesterday, at a time when the patient was laboring under a paroxysm of dyspnœa, there is evidently a less degree of enlarge-

* Although this contraction with inspiration is sometimes real as shown by one of these cases, in this, as in other instances, it is only apparent, and is occasioned by contraction of the intercostal spaces without contraction of the ribs.

ment. This is shown by the fact that then the mammary gland did not project, while now it is somewhat pendulous.

No heart impulse is discoverable, either in epigastrium or precordia.

The circumference over the chest, at the third rib, in expiration is 29 inches; in inspiration, 29¼ inches. Below the mamma the circumference is 27¼ inches. The latter remains unaffected either by inspiration or expiration.

No intercostal depressions are visible in front. They are slightly apparent at the inferior and lateral portion of the chest.

The percussion sound is very clear, in front on both sides. Relatively it is less clear, and the pitch is raised in the right infra clavicular region. The sense of resistance is greater on the right side. Over the upper part of the middle third, percussion sound equal on both sides. There exists relative dullness over the right scapula, and over the whole of the right side posteriorly.

On the left side, in front, with inspiration, there is a fine mucous râle, and with expiration a combination of sibilant and sonorous râles, the expiratory sounds much prolonged. On the right side still louder sibilant râle with prolonged expiration. Behind, a short rough sound of inspiration, and prolonged expiration with sonorous râle.

Slight vocal resonance over the right scapula, and none over the left.

Sounds of heart heard distinctly in precordia, and pure.

*Treatment.*—Nutritious, but non-animal diet, Stokes' linament to chest, anodyne expectorant mixture.

Dec. 15. The condition of the patient remaining about the same. Balsam of copaiba, in mixture, was prescribed, and no other remedies.

January 8, 1854. The patient reports better than at the date of the last record. The cough is less, and the expectoration much diminished. She has less habitual difficulty of respiration. Does not now experience much inconvenience except on exercise. The aspect is improved. The prolabia are dull in color, but not distinctly livid. The respirations are obviously performed with less labor. They are twenty per minute. The expirations are longer than the inspirations. No radial pulse is appreciable. Counted at the carotid artery. The pulse is one hundred per minute. She sits up all day. The appetite is good, and the bowels are regular. She feels quite weak. Respiration becomes laborious and attended with dyspnœa on exercise. She suffers from palpitation on exercise. This is not a new symptom although not previously recorded. When tranquil, the impulse of the heart is not preceptible, but after walking once slowly across the room it is felt over the whole precordia, the action accelerated, and the respirations are panting.

The expansive movement at the apex of the chest, as near as can be determined by the graduated tape, is from $\frac{1}{8}$ to $\frac{1}{4}$ of an inch. At the lower part there is neither contraction nor expansion.

On both sides loud sibilant râle with the expirations, and no murmur of inspiration.

Percussion sound notably clear, with tympantic quality.

No intercostal depressions visible at the upper part of chest.

The balsam of copaiba is continued.

The patient remains at the hospital at the time of writing this report (Jan. 23, 1854.)

---

CASE 3.—*John Scieller*, aged thirty-three, German, baker, admitted Dec. 31, 1853.

*Previous History.*—Patient states that his father was affected as he is, and died at the age of fifty-six years, as he thinks, from the same affection. He has brothers and sisters, none of whom have been similarly affected.

He recollects that when not more than six years of age he could not take active exercise without difficulty of breathing; and he has ever since experienced the same difficulty. It has uniformly been worse in the winter than in the summer. Otherwise his general health has been good.

Three weeks ago he fell overboard from a steamboat, and being unable to swim, was nearly drowned before being rescued. He was unconscious when taken out of the water. He was not confined to the bed immediately after this occurrence, but worked his passage on the boat up the river to this city. He worked a few days after arriving at Louisville, but was finally compelled by debility, cough and difficulty of breathing, to come to the hospital. A very satisfactory account of the previous history cannot be obtained, partly in consequence of the embarrassment of respiration rendering it difficult for him to talk, but still more from his imperfect knowledge of the English language.

*Present condition.*—Face presents a puffy and dingy appearance. Expression denotes distress. Appetite tolerable. Considerable thirst. Complains of headache. Bowels regular. Pulse 124, very small and full, pulsations occasionally lost. Extremities cold. Marked capillary congestion. Paroxysms of coughing frequently occur, with small expectoration. In these paroxysms the face becomes deeply congested and livid. He preserves a semi-recumbent posture. The respirations are 36 per minute, and greatly labored. The inspirations are short and spasmodic, and accompanied by dilatation of the *alœ nasi.* The expirations are at least four times as long as the inspirations.

*Physical Signs.*—The chest, in front, appears full and rounded, over and above the mammary region. The right side is more prominent than the left. No intercostal depressions visible except at the lower part of the chest, and here chiefly in the act of inspiration. The expansive movement with respirations is slight. The chief movement is that of elevation. The whole chest in inspiration is moved upward as in one piece. At the lower part the chest appears to contract with the inspirations. Above the sternum the integument collapses with the inspirations.

The percussion sound, on both sides, is notably clear, with somewhat of the tympanitic quality. This quality is more marked on the right side, and the pitch is higher.

In front, a respiratory sound is not uniformly appreciable. A faint, fine mucous râle sometimes heard on the right side at the end of the inspiratory act, and frequently a prolonged sibilant râle with the expiration. Behind, a clear percussion sound on both sides. At the upper part of the chest an indistinct murmur at the end of the inspiratory act.

A clear percussion sound in precordia, but the pitch higher than in corresponding region on the right side.

Heart sounds very feeble, and no impulse appreciable.

January 2, 1854. Since the previous date the patient has progressively improved. The respirations are now but moderately labored. They are twenty per minute. The alæ dilate slightly and the inspirations are still shortened. The countenance does not express suffering. The violent spasmodic cough has nearly disappeared. He now rarely coughs, and the quantity of expectoration is quite small. Appetite improving. Bowels regular. Pulse one hundred, and tolerably developed. The upper extremities present marked capillary congestion, and this exists in a moderate degree over the whole body.

During the severity of the symptoms, since the previous record, the patient exhibited delirium by incoherent talking.

The treatment pursued in the case has consisted solely of Hoffman's anodyne, ʒs three times daily, and whisky, ℥ss three times daily.

*Physical Signs.*—The chest, in front, still appears expanded; more so on the right, than of the left side. There are no intercostal depressions at the upper part of the chest; and at the lower part, in front and behind, they are only visible in the act of inspiration. They are then drawn in so as to be deeply marked. The whole chest moves, upward, as in one piece, with inspiration. The expansive movement, as determined by inspection, is but slightly apparent at the upper part of the chest, while the lower part appears to contract with inspiration. The expansive movements, determined as correctly as possible by means of the graduated tape, are as follows:—At the lower part of chest, with inspiration, the chest contracts about $\frac{1}{8}$ of an inch. Through the line of the nipple the chest is nearly immoveable; there is a very slight expansion. At the upper part, the expansive movement is about $\frac{1}{8}$ of an inch.

The percussion sound is very clear—drum-like, or tympanitic—with a ringing note. These characters are more marked on the right side. It is not easy to determine a marked variation in pitch, but on the left side the note seems somewhat higher.

No sound of respiration is appreciable anywhere either in front or behind. Nor is there either vocal resonance or fremitus.

Jan. 7. Patient reported feeling well enough to leave the hospital and he was accordingly discharged.

The foregoing cases afford excellent illustrations of the symptomatology, physical signs, and the historical events pertaining to emphysema of the lungs. The disease is one of those of which little was known prior to the minute investigations in morbid anatomy to which medical science is so much indebted for its progress during the past half century. The researches of LAENNEC led to the first distinct appreciation of the character and importance of the affection; so that, in the language of ROKITANSKY, "had LAENNEC done nothing else for medical science, his discovery of this diseased condition, and of the causes giving rise to it, would have sufficed to have rendered his name immortal." The disease still offers scope for study. There are points relating to its symptoms, signs, pathology, causation, etc., not yet fully settled, with reference to which accumulated clinical observations are to be desired.

In each of the three cases the symptoms denoting the commencement of the disease were traced back to early life; in one case as early as six years of age, in another at seventeen, and in the remaining case at sixteen. In two of the cases the disease had existed for many years; in the remaining case it was comparatively recent, having existed only two years.

In but one of the cases could hereditary influence be suspected to be concerned in the production of the disease. That a predisposition to it may be transmitted, was established, some years ago, by statistical researches prosecuted by the late JAMES JACKSON, jun., of Boston, Massachusetts.

In one of the cases the disease appeared to have its origin in an attack of measles. In the two remaining cases no circumstances appear in the previous history bearing on the causation except that the patients seem to have suffered from asthmatic paroxysms, and more or less cough and expectoration from the time that the existence of a constant difficulty was evidenced in habitual want of breath for active exercise. The connection which these symptoms may have with the origin of the disease will appear presently.

The causes of emphysema, and the mechanism of its production, are not, as yet determined satisfactorily to all minds. LAENNEC attributed the disease to a catarrhal affection seated in the smaller bronchial tubes, occasioning an obstruction, not sufficient to prevent the air from being forced into the cells by the act of inspiration, but offering a resistance to the egress of air from the cells with the less powerful act of expiration. This theory is now generally ignored, but a better explanation of the origin of a certain proportion of the cases of the disease has not been substituted. It seems reasonable that an incomplete obstruction, from any cause, at the smaller branches of the bronchial tree, in view of the relative power of the acts of inspiration and expiration, should lead to over-distension, and, at length, permanent enlargement of the cells. LAENNEC, however, supposed that the obstruction was due to solid sputa becoming impacted in the tubes. It is more probable that the swelling of the membrane is the source of the obstruction—that the affection is the result of subacute, or chronic capillary bronchitis, the minute bronchial tubes not being sufficiently obstructed to occasion the notable and dangerous embarrassment of respiration which characterises the acute form of that variety of bronchial inflammation. Bronchitis extending to the smaller tubes, accompanied with the formation of solid mucus, is more likely to lead to complete obstruction; and, in consequence of the obstruction being complete, to collapse of the air vesicles, and pulmonary lobules, rather than emphysematous enlargement. It may at first strike the mind of the reader as paradoxical thus to attribute two lesions directly opposite in their character, viz: emphysema and collapse, to the same, pathological source, viz: capillary bronchitis. But a little examination of the subject will serve to explain the apparent inconsistency. In certain cases of bronchitis in which the obstruction is due simply, or mainly, to the swelling of the membrane, and is incomplete, the air finds access to the cells in consequence of the power of the inspiratory act being sufficient to carry it there in spite of the narrowed passages; but it is not readily expelled from the cells, because the obstacle afforded by the narrowed tubes is too great to be fully overcome by the relatively weaker act of expiration. As a necessary result, over-distension of the cells ensues, and, if sufficiently continued, emphysema, or permanent enlargement of the cells, takes place. On the other hand, if, in addition to the diminished calibre of the bronchial tubes, they are obstructed by plugs of mucus, there follows total obstruction in a portion of the tubes to the entrance of air into the cells *with inspiration.* The obstacle is now especially present in inspiration, this act forcing the plugs of mucus into the smallest extremities of the tube. But in expiration the force of the air already within the cells tends to remove the mucus from the smaller, to the larger tubes, and the resistance is therefore much less, and perhaps but slight. As a consequence of this less, or slight obstruction *in expiration*, the cells empty themselves of their contained air, and become collapsed, the total obstruction in inspiration, preventing them from becoming refilled. The plugs of mucus, in short, act precisely as *ball valves*, opposing the passage of air *into* the cells; but permitting its escape *from* the cells. Under these circumstances the lesion directly opposite to that of emphysema occurs, viz: collapse.

A late writer, Dr. W. T. Gardener, accounts for the production of emphysema by regarding it as a secondary result of collapse of more or less of the pulmonary lobules.* He supposes, that, in consequence of obstructions in the bronchial tubes, collapse of the cells takes place in the manner just described, and, as a consequence of this collapse, an additional burthen being thrown on the non-collapsed vesicles, the latter become over-distended, or emphysematous. It is perhaps sufficient to say with respect to this theory, that, were it correct, in cases of chronic pleurisy in which the entire lung on one side is compressed into a small solid mass, we should expect emphysema of the lung on the side unaffected with pleurisy to be an uniform result; and it is certainly not true that chronic pleurisy leads, thus, to emphysema.

That chronic bronchitis is involved in the production of emphysema is rendered probable by the fact of the co-existence of the two affections very frequently, if not generally, in cases of the latter. In the three cases reported in this article, more or less bronchitis, evidenced by cough and expectoration, had existed from the date of the symptoms of the emphysema. Now, it is more reasonable to suppose, if there be a relation of cause and effect between the two affections, that the emphysema is due to the bronchitis, than that the bronchitis supervenes on the emphysema. With reference to this subject it would be desirable to determine in a series

* Edinburg Monthly Journal of Medical Science.h of cases which affection took precedence of the other, or in how large a proportion of instances chronic bronchitis existed during the development of emphysema. Information, as positive as is to be desired, on these points is not easily obtained, inasmuch as, in most of the cases coming under observation, the affection has already existed for a long time, and the memory of the patient with respect to the circumstances attending its development may not be altogether reliable. Moreover, the affection, in a large proportion of instances, is developed gradually, and the earliest symptoms extend backward to youth, or childhood.

But bronchitis is not the only morbid condition involving a degree of obstruction not sufficiently great to deprive the cells of inspired air, but sufficient to keep the cells distended by interfering with the expulsion of the air from the cells by expiration. Spasm of the muscular fibres entering into the composition of the bronchial tubes will lead to the same result. The latter is the morbid condition constituting pure *Asthma.* So soon as emphysema began to be studied, it was found that it was very frequently associated with asthma. These two affections evidently sustain to each other some close relation. The opinion held by LOUIS, and others, is, that asthma is incidental to the emphysema, but there is good reason for believing that the reverse of this is true, viz: that the emphysema is the effect of asthma. In the cases now reported, asthmatic paroxysms occurred from the first evidences of pulmonary disorder, and this is probably the fact in the history of the disease in a large proportion of cases. We can understand why repeated attacks of asthma should induce emphysema. The mechanism is the same as when it is due to bronchitis without spasm. Moreover, bronchitis and spasm are frequently associated; or more correctly, spasm is often excited in connection with bronchial inflammation, although the latter is by no means necessarily involved. On the other hand, no reason can be assigned why the existence of emphysema should give rise to spasm, although, of course, in proportion to the former lesion, the distress incident to the latter condition will be enhanced. In pure asthma the lung necessarily is in an emphysematous state, temporarily, during the continuance of the paroxysm—that is to say, the cells are distended. This distension, by frequent repetition has only to become permanent, to constitute the lesion. On this question the following extract from a clinical lecture by M. TROUSSEAU, which has recently fallen under notice, is in point:—*

"An illustrious man, M. LOUIS, has confounded emphysema of the lung with asthma, attributing the latter to the organic pulmonary lesion. This opinion will not bear a minute examination, and the cause of its error is easily found. When M. LOUIS has examined old asthmatics in hospitals, he has found pulmonary emphysema. Not in his private researches, in young men, suffering from asthma, whom I have sent him, who have been examined by eminent men of art, has he been able to find a trace of emphysema. M. LOUIS is right when he affirms that emphysema exists after asthma, but he is wrong when he makes it precede that disease."

Waiving farther remarks (which have perhaps already been too extended) on the causation of emphysema, I proceed to give a brief summary, *First*, of the distinctive events belonging to the symptomatology of the disease; and, *secondly*, a *résume* of the physical signs.

Habitual labor of respiration characterises the affection, the amount of effort manifested in the respiratory acts being commensurate with the degree and extent of the lesion. The labor is especially marked in *expiration.* This is a distinctive feature The difficulty is in expelling the air from the cells. When this difficulty is great the abdominal muscles are made instinctively to coöperate with the diaphragm in the endeavor to compress the lungs as much as possible.

The rhythm of the respiratory acts is altered, the expiratory act becoming longer than the inspiratory, and this inversion in the relative duration of the two acts is proportionate to the degree of the lesion. The relatively prolonged expiration is due, not alone to an actual prolongation of the expiration, but to shortening of the inspiration, and the lesion induces the latter just in proportion as it involves the former.

Anything which tasks unusually the respiratory function occasions increased labor, amounting, in severe cases, to paroxysms of dyspnœa of greater or less severity. Running, walking, especially up an acclivity, (as going up stairs,) or any active exercise, pro-

* Gazette des Hôpitaux, Paris. Virg. Med. and Surg. Journal.

duces embarrassment proportionate to the lesion.* After a time, however, this, and other symptoms, are due, not altogether to the pulmonary lesion, but to the enlargement of the heart incidental to the lesion, the latter being produced by the obstruction to the pulmonary circulation incident to the lesion. The enlargement of the heart, occurring as the result of emphysema, commences with the right ventricle, for the obvious reason that the accumulation of blood consequent on the pulmonary obstruction first takes place in this cavity.†

The occurrence of bronchitis, however mild, and even pulmonary catarrh, are accompanied by notable disturbance of the respiration; which, also, is more or less distressing according to the degree and extent of the emphysema. Bronchitis, even if severe, except in the rare variety in which the inflammation affects the capillary tubes, as is well known, does not occasion embarrassment of respiration, if the lungs are otherwise sound. The explanation of the suffering which it occasions in the emphysematous state is obvious. Emphysema compromises the capacity of the lungs for respiration in the modes already mentioned. In consequence of this lessened capacity, the patient when free from bronchitis, or any superadded affection, is compelled to use the organs fully, to their utmost ability, and with more or less labor, in ordinary respiration. Now, if the lungs are sound, in ordinary respiration, all the air vesicles are not expanded. In other words, a healthy person has more lung capacity than he requires for ordinary use. He has a superfluity of cells, which are held in reserve for any emergency. Bronchitis involves more or less tumefaction of the bronchial mucous membrane, and consequent diminution of the calibre of the bronchial tubes. But this is not felt, inasmuch as a portion of the reserved cells are brought into use to compensate

*In a case which came under the writer's observation, the patient complained that he could not laugh heartily without losing his breath and suffering from dyspnœa. Deriving much of his enjoyment from telling, and listening to good stories, he felt this inability to be one of the most serious of the inconveniences attending the disease.

†DR. WALSHE states that the number of respirations per minute is not increased, but diminished in emphysema. This is probably true of some cases, but that it is not the rule, is shown by the cases reported in connection with these remarks.

for the contraction of the bronchial tubes; a little more force of inspiration is instinctively employed, and no dyspnœa is experienced, nor any evidences of difficulty manifested. On the contrary, the emphysematous patient affected with bronchitis, has no reserved capacity of lung for this, or any other emergency. The organs in his best estate being taxed to the extent of their ability to perform the function of hematosis, whenever the supply of air is diminished the want of breath is felt, and the symptoms incident to dyspnœa are present. The effect of bronchitis occurring in connection with emphysema is illustrated in each of the three cases reported, especially in the last of the three cases, the difficulty of respiration in that case being very great and apparently placing the life of the patient in danger. If I have succeeded in making this explanation clear, it will be understood why it is that bronchitis is a very different affection when it is associated with a considerable amount of emphysema, from what it is when it occurs in a healthy person.

Capillary congestion most marked on the face; next, on the upper extremities, and more or less over the whole body, is a symptom, unless the lesion be slight. This symptom is proportionate to the secondary heart affection, and its presence in a marked degree is one of the evidences of the latter complication. The symptom is especially marked during paroxysms of difficult respiration, and may then, and in some instances habitually, be conjoined with more or less lividity of the prolabia.

The expression of the patient is anxious, and denotes distress in proportion as the respiration is laborious, and the sense of breathlessness felt. It will, however, often be observed that, owing to the influence of habit, patients are not conscious of the respiratory effort which is apparent to others. It is remarkable that persons in whom the lesion exists in a degree to cause habitually labored respiration, enlargement of the heart also existing, manage to continue in occupations requiring considerable muscular exertion. This was true in the first and third of the cases reported in connection with these remarks.

Smallness and feebleness of the pulse characterize the affection, and these characters are more marked in proportion to the amount of heart complication.

Coolness or even coldness of the surface is another symptom, bearing some relation to the emphysema, but more to the consecutive disease of heart.

As in all affections involving difficulty of respiration, the embarrassment is greater in the night, than in the day time; and paroxysms of dyspnœa oftener occur during the night.

Paroxysms are excited by any thing overtasking the respiratory function, but they are especially due to catarrh, bronchitis, and spasm of the bronchial muscular fibres. The odor of feathers, which provokes attacks of asthma in some persons who are not subject to that disorder under other circumstances, in one of the cases reported, always occasioned insupportable dyspnœa.

More or less cough and expectoration, certainly in many cases of emphysema, are habitual; and the acts of coughing are characteristic. The coughing is violent, spasmodic, resembling, as stated in the notes of one of the reported cases, pertussis exclusive of the sonorous inspiration. The existence of cough and expectoration, conjoined with the difficulty of determining by means of physical signs in this affection, the presence of a tuberculous deposit, would lead us to have apprehensions of the latter oftener than we do, had not statistics determined that tuberculosis and emphysema are not often associated. The state of the lung in emphysema appears to exert a protective influence against the deposit of tubercle.

The symptomatology of emphysema is quite significant in a diagnostic point of view, but taken in connection with the physical signs, the discrimination of the disease is positive and easy. *Inspection* furnishes some characteristic appearances. In the variety of emphysema characterized by increased volume of the lungs, the chest is permanently distended. This enlargement is general, or partial, according as the emphysema affects the whole, or a portion of the pulmonary organs. It is rare that lesion is universal, and it is seated at the upper portion of the lungs in the great majority of cases. The prominence is then marked in the supra clavicular and infra clavicular regions. The parts here may not only be prominent, but bulging. It is rare that the lungs on both sides are affected in an equal degree, and the lesion may be limited to one side. In the three cases now reported the upper portion of the lungs on both sides were especially affected, and on one side more than the other, the latter is generally the fact. It is very rarely the case, if ever, that the lesion is equal in degree on both sides. In two of the cases the greater amount was on the right, and in the other case on the left side.

The expansive movement with inspiration is limited, becoming sometimes almost null. The chest is permanently expanded. The increased volume of the lungs resists the forces by which the contraction of the chest in expiration is produced. Hence, of necessity, the expansion is correspondingly diminished. The elevation movement is alone conspicuous, and this, especially in a paroxysm of labored respiration, becomes marked. The chest, in the language of Dr. WALSHE, already quoted, "moves upward as in one piece." Owing to the forcible contraction of the diaphragm acting upon the false ribs, the lower part of the chest appears to contract with inspiration. This is apparent sometimes, not real, owing to the intercostal spaces being drawn inward, exclusive of the ribs, but the latter, in some instances, are also contracted, as was exemplified in one of the reported cases.

Different observers express different opinions respecting the permanent effect on the intercostal spaces. Dr. STOKES stated that the normal depressions between the ribs are preserved in this disease. This may be true, as remarked by Dr. WALSHE, when the emphysema is of the atrophous kind, and the volume of the lungs is not increased. It is certainly not true of cases characterized by a marked increase in the volume of the lungs, as was illustrated in each of the cases now reported. A positive statement on this point, even in opposition to so high an authority as Dr. STOKES, is the more admissible, inasmuch as the matter is one of simple clinical observation.

If the lesion be extensive, affecting both sides, the form of the chest is notably altered. The anterior flattening is lost. The thorax, as stated by ROKITANSKY, becomes *barrel-shaped*, the ribs pursue a less oblique direction, approximating to a horizontal line, and the same change is observed, after death, in the direction of the fissures dividing the pulmonary lobes.

*Percussion* elicits an abnormal degree of resonance. The vesicular quality is not lost, so that the sound is readily distinguished from the tympanitic sonoriety transmitted from the abdomen, or due to air in the pleural cavity, in pneumo-thorax. The increased proportion of air to the tissues, and the tension of the thoracic walls causes an admixture of tympanitic and vesicular resonance, giving to the percussion note a ringing, drum-like character. This modification, and the abnormal sonoriety will be relatively more marked, of course, on the side in which the emphysematous distension is greatest.

The signs determined by *Auscultation*, taken in connection with those obtained by the foregoing methods, are distinctive. If the patient, at the time of the examination, labor under bronchitis, the bronchial râles are usually extremely loud, especially the sonorous and sibilant râles. They are frequently heard at a distance. They may accompany both acts of respiration, but they are louder and longer with the act of expiration, and sometimes limited to this act.

When the conditions producing the bronchial râles are absent, the respiratory murmur, if present, is characterized, *first*, by an abnormal diminution in intensity. It may be inappreciable, as in the third of the reported cases, after the patient had recovered from the attack of bronchitis. If appreciable, a *second* modification consists in an alteration of the rhythm. The inspiratory sound is shortened, and is heard only at the end of the inspiratory effort. The expiratory sound is prolonged, exceeding, by several times, the sound of inspiration. The inspiratory sound may be absent, and the expiratory, only heard; or, on the other hand, the only sound heard may be a shortened, feeble, inspiratory murmur. The change in rhythm, first pointed out, is analogous to that occurring both in pneumonitis, and the stage of crude tuberculosis, due in these affections, to solidified lung. This analogy, however, cannot lead to error of diagnosis. The bronchial respiration of pneumonitis and tuberculosis has other characters, viz: elevation of pitch of sound, the expiration higher in pitch than the inspiration, and, usually, greater intensity in both the inspiratory and expiratory sounds.* Moreover, the associated signs, viz: dullness or flatness on percussion, (the evidences of solidification) are in direct contrast with the abnormal resonance in emphysema.

* See prize Essay by writer.

Bronchophony and vocal fremitus, in emphysema, are either wanting, or slight. These signs, also, are generally marked in diseases involving solidification.

To determine, by means of physical signs, the existence and degree of the enlargement of heart incident to this disease, is much more difficult than to make the diagnosis of emphysema. If the emphysema be extensive, and affect the left side, the heart is overlapped by the overdistended lung, and removed from its normal situation. An impulse is frequently not to be felt. The increased sonoriety of the chest, and the disparity, in this respect, of the two sides, owing to difference in the degree of emphysema, interfere with the attempt to define the boundaries of the organ by percussion. We are to be guided, in forming an opinion with respect to this complication, in a great measure by the symptoms. Palpitation, if a prominent symptom, is strong evidence, but by no means conclusive. I have known this symptom to be so prominent in a case of senile emphysema, that the attending physician supposed the patient to labor under a heart affection, and this alone. I was desired to make an autopsy after the termination of that case, with a view to the discovery of heart disease, but there proved to be a very remarkable degree of atrophous emphysema, which had been rapidly developed, and the heart was sound. Great feebleness of the pulse, amounting sometimes to extinction at the wrist (as in one of the reported cases), marked capillary congestion of the surface; and especially lividity, in connection with palpitation, render it pretty certain that more or less enlargement of heart co-exists.*

Emphysema is an incurable lesion. If atrophy and dissolution of the cell walls have taken place, either as a point of departure for the disease, or consecutively to dilation, it is sufficiently clear that so far as this is concerned, restoration is impossible. Lung

---

* The reader will bear in mind that in this account of physical signs I have had reference particularly to emphysema, characterized by increased volume of the lungs. In emphysema commencing with atrophy, and not accompanied by marked increase of volume, the physical signs would not be in all respects the same. The differences as respects enlargement of the chest, less sonoriety on percussion, and depression of the intercostal spaces (to the latter, allusion has been made,) will at once suggest themselves. As regards the more distinctive of the physical signs, and symptoms, the two forms of the disease are identical.

structure cannot be reproduced. But so far as the lesion involves dilatation of the cells it is by no means certain that, although a complete cure is not to be expected, a considerable diminution of the lesion may not take place. It is obvious that the management of the disease does not embrace any special medication. We cannot expect that any remedy will act upon the lungs to obviate directly the effects of a mechanical agency—for the mode by which the dilatation of the cells is produced is mechanical. The curative indications relate to, *first*, removing, if possible, the morbid conditions which produce and perpetuate the lesion. We have seen that bronchitis and spasm are the morbid conditions which especially stand in the relation of causes to it. So far as we can control these conditions we are able to prevent the development, and limit the progress of emphysema. *Second*, It is important to avoid every thing which overtasks the respiratory function, and occasions temporary repletion of the cells, evidenced by labored respiration, or dyspnœa. Occupations requiring muscular effort, and active exercise of any description, must tend to confirm and increase the lesion. So any thing interfering with the respiratory function, as the impure atmosphere of crowded rooms, or the inhalation of irritating substances, will have a similar effect. Excitement of the circulation by the use of stimulants, or by other cause, will, measurably, do injury in a similar manner.

It is foreign to the purpose of this article, to consider, in detail, the measures which may be involved in fulfilling these indications; and in connection with remarks addressed to the practitioner of medicine this would be superfluous.

In dismissing the subject, the following practical consideration is submitted as one which should commend itself to the thoughts of the medical practitioner: In view of the probability that, in many instances, before emphysema has advanced to an extent leading, necessarily, to distressing and serious consequences, judicious management may greatly retard, if not arrest its progress, it is a matter of great importance, in the first place, to appreciate the pathology and causation of the affection so far as our existing knowledge enables us to study the lesion in these aspects; and, in the second place, to be familiar with the symptoms and physical signs with reference to an early and positive diagnosis.

